# Comparison of the properties of polyimide nanocomposite films containing functionalized-graphene and organoclay as nanofillers

**DOI:** 10.1038/s41598-022-25178-2

**Published:** 2022-12-03

**Authors:** Moon Young Choi, Seon Ju Lee, Ae Ran Lim, Jin-Hae Chang

**Affiliations:** 1 Graduate School of Carbon Convergence Engineering, Jeonju University, Jeonju, 55069 Korea; 2 Department of Science Education, Jeonju University, Jeonju, 55069 Korea; 3 Institute of Carbon Technology, Jeonju University, Jeonju, 55069 Korea

**Keywords:** Materials science, Polymer chemistry

## Abstract

Poly(amic acid) (PAA) is prepared by the reaction of dianhydride 4,4′-biphthalic anhydride and diamine bis[4-(3-aminophenoxy)phenyl]sulfone in *N,N’*-dimethylacetamide. Two types of fillers were dispersed in the as-synthesized PAA via a solution intercalation method; polyimide (PI) hybrid films were synthesized under various heat treatment conditions. Octylamine (C8) was introduced into graphene sheets (C8-GS) and bentonite (C8-BTN), which were then used as nanofillers in the PI hybrid films. The synthesized nanofillers were used in varying amounts of 0.25–1.00 wt% with respect to the matrix PI. The thermal and morphological properties and optical transparency of the hybrid films were investigated and compared for both C8-GS and C8-BTN at varying nanofiller content. The C8-BTN nanocomposite showed superior thermal properties, and optical transparency, and the filler was well dispersed in the PI matrix compared to the C8-GS nanocomposite. The thermal stability of the hybrid films improved upon the addition of small amounts of the nanofiller. However, beyond a certain critical filler concentration, the thermal stability declined. These results were verified through the dispersion of fillers via transmission electron microscopy.

## Introduction

Aromatic polyimides (PIs) are one of the most important super-engineering materials owing to their excellent mechanical properties and thermal stability at high temperatures^1,2^. In addition, PIs are easy to synthesize, can be made into thin films, and do not require crosslinking for curing owing to the presence of imide bonds in the polymer backbone. Several recent reports involve the integration of PIs into semiconductor materials, such as liquid crystal displays (LCDs) and plasma display panels (PDPs), because of their light weight and the precision of electronic products^3–5^.

Weight reduction and miniaturization of displays have become important. Because the currently used glass substrate is heavy and brittle and difficult to process continuously, the application of PIs in mobile phones, laptops, and personal digital assistants (PDAs) is being actively researched^6–8^. Further, many studies focus on the application of PIs in light and flexible plastic displays, which overcomes the disadvantages induced by the use of glass substrates^9,10^. Despite the advantages of PIs in various fields such as electronics, it is used limitedly in transparent flexible printed circuit boards and displays because of their inherent dark brown color^11,12^.

The brown color of PIs can be explained by the charge-transfer complex (CTC) theory, in which the π electrons of benzene in the main imide chain interact via intermolecular bonding. To synthesize colorless and transparent PIs (CPIs), the charge-transfer interactions should be lowered, i.e., the movement of π electrons should be restricted. This can be achieved by introducing strongly electronegative functional groups, such as trifluoromethyl (–CF_3_), sulfone (–SO_2_), and ether (–O–), into the main chain. Alternatively, a whole curved backbone structure may be used^13–15^.

Currently, organic polymers such as polycarbonate (PC), poly(ethylene terephthalate) (PET), and poly(methyl methacrylate) (PMMA) are mainly used in electro-optics. They exhibit a transmittance of ≥ 80% at 500 nm. Despite the favorable optical properties, their glass transition temperature (*T*_*g*_) is less than 150 °C. Thus, they are unsuitable for use in electronic devices manufactured at high temperatures. Recently, attempts have been devoted toward the substitution of such polymers with CPIs that have excellent thermal stabilities^16,17^.

Layered polymer nanocomposites obtained by the exfoliation of polymer matrix fillers, such as clay or graphene, are versatile materials, which exhibited performance superior to that of simple polymer blends and composites. Synthetic fillers include carbon nanotubes (CNTs), graphene, polyhedral oligomeric silsesquioxanes (POSSs), and layered double hydroxides (LDHs), while clay and metal powders constitute natural fillers. Although graphene has excellent physical properties, it is difficult to process and expensive. Moreover, the dispersion of graphene in polymer matrices is complicated. This difficulty is gradually being overcome by a recently developed method^18–21^. Many studies have been conducted on clay and graphene. In particular, clay has been used for a long time because it is inexpensive and naturally available.

When nanometer (nm) sized filler particles are uniformly dispersed in a matrix polymer, it not only improves various thermo-mechanical properties of the composite material but also shows excellent physical properties in terms of dimensional stability. In particular, if nanoparticles with a large aspect ratio (length/width) are perfectly dispersed in the polymer, the length and number of paths through which the gas passes increase. The gas barrier properties, heat resistance, solvent resistance, and insulation properties also increase^22–24^.

Most of the clays used as nanofillers have a layered silicate structure. Smectite-based clays with a layered structure generally comprise sheets of silica tetrahedra and aluminum octahedra in a 2:1 ratio^22^. Commonly used smectite clays include bentonite (BTN), montmorillonite (MMT), saponite, hectorite, and mica; BTN is mostly composed of MMT (~ 80%) with small amounts of other minerals, such as mica, calcite, illite, quartz, and chlorite. BTN is widely used in cosmetics, pharmaceuticals, paints, ceramics, and packaging materials, and serves as an effective nanohybrid filler owing to its unique dispersion, adsorption, and catalytic properties^23,24^. As is well known, graphene has excellent thermal conductivity, electrical conductivity, and very high mechanical properties along with CNTs. For example, single-layer graphene has a large surface area of 2630 m^2^/g, a thermal conductivity of 5000 W/mK, and an electrical conductivity of 6000 S/cm. Because of these properties, graphene has sufficient potential for use in the production of gas barrier materials, transparent electrodes, and solar cells, which are currently being extensively studied^25,26^.

However, most pristine clays, like graphene, show low dispersibility or compatibility in a polymer matrix, which degrades the physical properties of the composite material. To overcome these shortcomings, an organoclay or functionalized graphene obtained by including organic molecules into the clay or graphene can be used as an alternative filler. Such modified fillers can be dispersed in the polymer matrix in the nanoscale, without agglomeration or the consequent phase separation. Thus, such fillers can be used in the production of nanohybrid materials^27–30^.

Herein, a PI was synthesized using monomers 4,4′-biphthalic anhydride (BPA) and bis[4-(3-aminophenoxy)phenyl]sulfone (*m*-BAPS) as the dianhydride and diamine, respectively. These monomers exhibit a bent meta structure and include the highly electronegative –SO_2_– group in the polymer main chain, which render them with transparency and flexibility. Although many CPIs have halogen-containing moieties, e.g., –CF_3_, the as-synthesized PI is halogen-free, which makes it environmentally friendly.

In the precursor state, nanofillers were dispersed and eventually PI hybrid films were prepared through thermal imidization. The PI hybrid films were synthesized using varying amounts of two types of nanofillers, and the resulting effects on the thermal, optical, and morphological properties were investigated. In addition, the physical properties of the nanohybrid films obtained using two different fillers were compared. Organoclay (C8-BTN) was obtained by chemically modifying BTN with octylamine (C8), while a functionalized graphene sheet (C8-GS) was obtained by reacting C8 with graphene oxide (GO). For synthesizing the hybrid, the filler content was varied from 0.25 to 1.00 wt% with respect to the polymer matrix. To compare the filler effects of GS and BTN on the CPI matrix, the length of the organic moiety in chemically modified nanofillers was equal to that of C8.

As mentioned above, several studies have investigated the BTN and graphene nanofillers used in this study, and these studies have reported remarkable improvements to the physical properties of polymer nanocomposites using these fillers^23–26^. Although the color of the film of the composite material darkened when the concentration of graphene in the matrix polymer increased, the physical properties improved. In this study, the effect of the graphene content that can maximize the physical properties, while minimizing the dark color of the film, was also considered.

This study also involves varying the type and amount of the filler used in the preparation of PI hybrid films via the solution intercalation method. The influence of this variation on the morphology, thermal properties, and optical transparency of the films is explored, and the differences between the hybrid films obtained using two types of fillers are compared.

## Methods

### Materials

Monomers BPA and *m*-BAPS were purchased from TCI (Tokyo, Japan). *N,N’*-Dimethylacetamide (DMAc) and C8 were purchased from Junsei (Tokyo, Japan), and were used after complete moisture removal by passing them through a molecular sieve (4 Å). GO and BTN were purchased from Standard Graphene Co. (Ulsan, Korea) and Sigma-Aldrich (Yongin, Korea), respectively. The cation exchange capacity (CEC) of BTN was 82.3 meq/100 g^22,23^.

### Synthesis of C8-GS

The C8-GS filler was synthesized from GO. In a 100 mL beaker, 0.20 g (1.55 × 10^−3^ mol) of C8 was dissolved in 25 mL of ethanol and stirred for 30 min. In a separate 250 mL three-necked flask under N_2_, 0.10 g of GO was added to 150 mL of distilled water and stirred for 30 min. The two solutions were mixed at 25 °C for 16 h. The resulting precipitate was stirred for 30 min in 1:1 v/v aqueous ethanol at 25 °C, and subsequently washed and filtered. This process was repeated 2–3 times. The obtained solid product was dried at 70 °C for 24 h (81% yield).

### Synthesis of C8-BTN

To a solution of 1.92 mL concentrated HCl in 100 mL of distilled water, 1.23 g (9.51 × 10^−3^ mol) of C8 was added and stirred for 1 h under N_2_ at 80 °C. In a separate beaker, 100 mL of distilled water and 4 g of BTN were mixed and stirred at 80 °C for 1 h. Subsequently, this solution was mixed with the C8 solution and stirred at 80 °C for 2 h. The white precipitate obtained was filtered and washed in 100 mL of distilled water at 25 °C for 1 h (repeated 2–3 times). The obtained precipitate was washed in a 1:1 (v/v) aqueous ethanol at 25 °C for 30 min. The obtained white solid (C8-BTN) was dried under vacuum at 25 °C for 24 h (86% yield).

### Synthesis of PI hybrid film containing C8-BTN

**Scheme 1:**
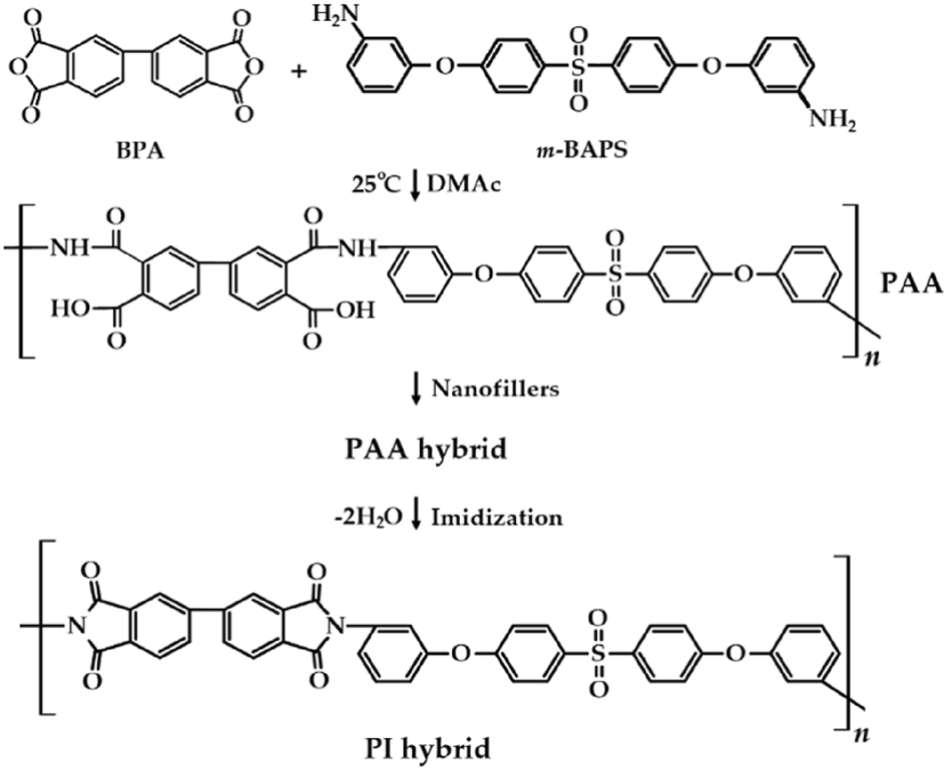
Synthesis route for the fabrication of the PI hybrid films.

The synthesis protocol of the hybrid films was the same regardless of the type and content of nanofillers. As an example, the preparation of the PI hybrid film using 0.50 wt% of C8-BTN included the following steps: In a 100 mL three-necked flask under N_2_, 3.84 g (1.3 × 10^−2^ mol) of BPA was dissolved in 30 mL of DMAc and stirred at 25 °C for 30 min. In a separate flask, 5.62 g (1.3 × 10^−2^ mol) of *m*-BAPS was dissolved in 30 mL of DMAc and stirred at 25 °C for 30 min. The two solutions were mixed and reacted at 25 °C for 16 h to synthesize a poly(amic acid) (PAA) solution. Subsequently, 0.05 g of C8-BTN was mixed with 50 mL of DMAc and dispersed for 3 h. The dispersed BTN solution was mixed with the PAA solution and stirred at 25 °C for 30 min. The stirring was continued for an additional 90 min at room temperature to stabilize the PAA solution. Subsequently, the PAA solution was poured on a 10 cm × 10 cm glass plate and the final PI hybrid film was synthesized using various heat treatment conditions (Table 1) under vacuum. The overall hybrid synthetic method is shown in Scheme 1.

**Table 1:** Heat treatment conditions of the PI hybrid films.

Samples	Temperature (°C)/time (min)/pressure (Torr)
PAA	50/120/1 → 80/60/1
PI Hybrid	110/30/1 → 140/30/1 → 170/30/1 → 195/50/1 → 220/50/1 → 235/240/1

The thickness of the synthesized PI hybrid film varied from 40 to 50 μm depending on the content of the nanofillers. To obtain reliable results based on the content of the fillers, the thickness of the film was kept as constant as possible.

### Characterization

The thickness and surface morphology of the graphene layer were explored using an AutoProbe CP/MT scanning probe microscope attached with an atomic force microscope (AFM, Multimode, NanoScope III, Digital Instruments Inc. New York, NY, USA). After the sample was dispersed in the solvent, the solution was sonicated for 3 h, and spin-coated on a silicon wafer.

The structures of the synthesized nanofillers and PI were confirmed using Fourier-transform infrared (FT-IR) spectroscopy (PerkinElmer, Spectrum Two, Llantrisant, UK). A solid-state ^13^C cross-polarized magic angle spinning-nuclear magnetic resonance (CP/MAS NMR) spectrometer (Bruker 400 DSX NMR, Berlin, Germany) at a Larmor frequency of 100.61 MHz was used, and the chemical shift was based on the reference tetramethylsilane (TMS) signal.

Wide angle X-ray diffraction (XRD) analysis (PANalyticalX' Pert PRO-MRD, Amsterdam, Netherland) was performed to investigate the dispersion state and interlayer distance of the nanofillers in the matrix. The target was Cu-Kα; the measurement range was 2*θ* = 2°–15°, and the measurement speed was 2°/min. Electron microscopes were used to investigate the detailed morphology. The film was quenched in liquid N_2_ and then broken, and the film cross-section was examined via field emission scanning electron microscopy (FE-SEM) (JEOL, JSM-6500F, Tokyo, Japan). Transmission electron microscopy (TEM) (JEOL, JEM 2100, Tokyo, Japan) was used to investigate the dispersion morphology of the hybrid film, and after curing the specimen with epoxy to prepare a TEM sample, it was heated in an oven at 70 °C for 24 h. After applying a vacuum, a 90-nm-thick sample was prepared using a microtome equipped with a glass knife. The acceleration voltage used was 120 kV.

A differential scanning calorimeter (DSC, 2-00915, Delaware, USA) and thermogravimetric analyzer (TGA, SDT 0650-0439, Delaware, USA) were used to measure the thermal properties of the hybrid film; the analysis was conducted at 20 °C/min in N_2_. The coefficient of thermal expansion (CTE) values were obtained via a thermomechanical analyzer (TMA, SS6100, Tokyo, Japan). The size of the film was 5 mm × 30 mm, and the heating rate was 5 °C/min under an expansion force of 0.1 N. The CTE values were obtained from the results through secondary heating.

To investigate the mechanical properties, a universal tester (UTM, Model 5564, Instron, Seoul, Korea) was used. The sample size was 5 × 50 mm^2^ and the crosshead speed was measured at 2 mm/min. From the results obtained by measuring the sample more than 10 times, the results with severe errors were excluded and the rest were averaged.

To investigate the optical properties, the cut-off wavelength (λ_o_) and light transmittance in the visible range were measured using a UV–visible spectrometer (Shimadzu, UV-3600, Tokyo, Japan). The yellow index (YI) was measured using a spectrophotometer (Konica Minolta, CM-3600d, Tokyo, Japan).

## Experimental results

### FT-IR spectra of the nanofillers

**Figure 1:**
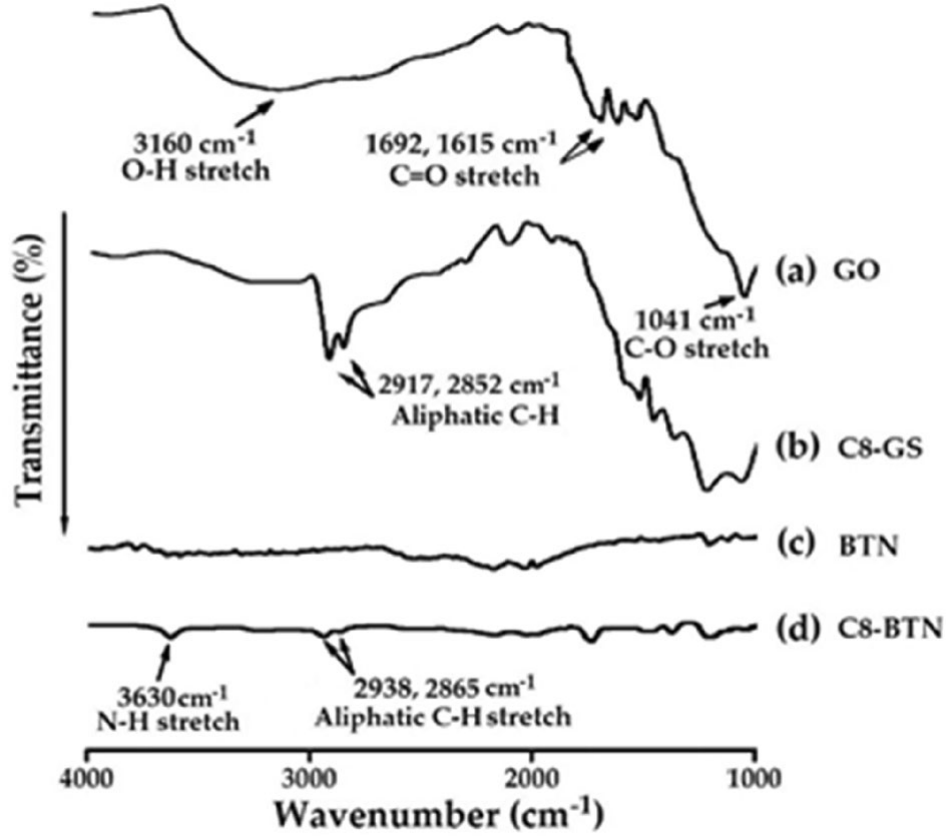
FT-IR spectra of (**a**) GO, (**b**) C8-GS, (**c**) BTN, and (**d**) C8-BTN.

Figure 1 shows the FTIR spectra of GO, BTN, and the organofillers chemically modified with C8 (C8-GS and C8-BTN). The spectrum of GO (Fig. 1a) shows a broad O–H stretching peak at 3160 cm^−1^ and C = O stretching peaks at 1692 and 1615 cm^−1^. Additionally, a C–O stretching peak at 1041 cm^−1^ is observed. In case of C8-GS (GO functionalized using C8) (Fig. 1b), the alkyl C–H peaks of C8 are observed at 2917 and 2852 cm^−1^. No peak is observed in the spectrum of pristine clay BTN (Fig. 1c), but for C8-BTN, N–H stretching peaks at 3630 cm^−1^ and aliphatic C–H stretching peaks at 2938 and 2865 cm^−1^ are observed (Fig. 1d)^31^.

### Functionalized GS: C8-GS

**Figure 2:**
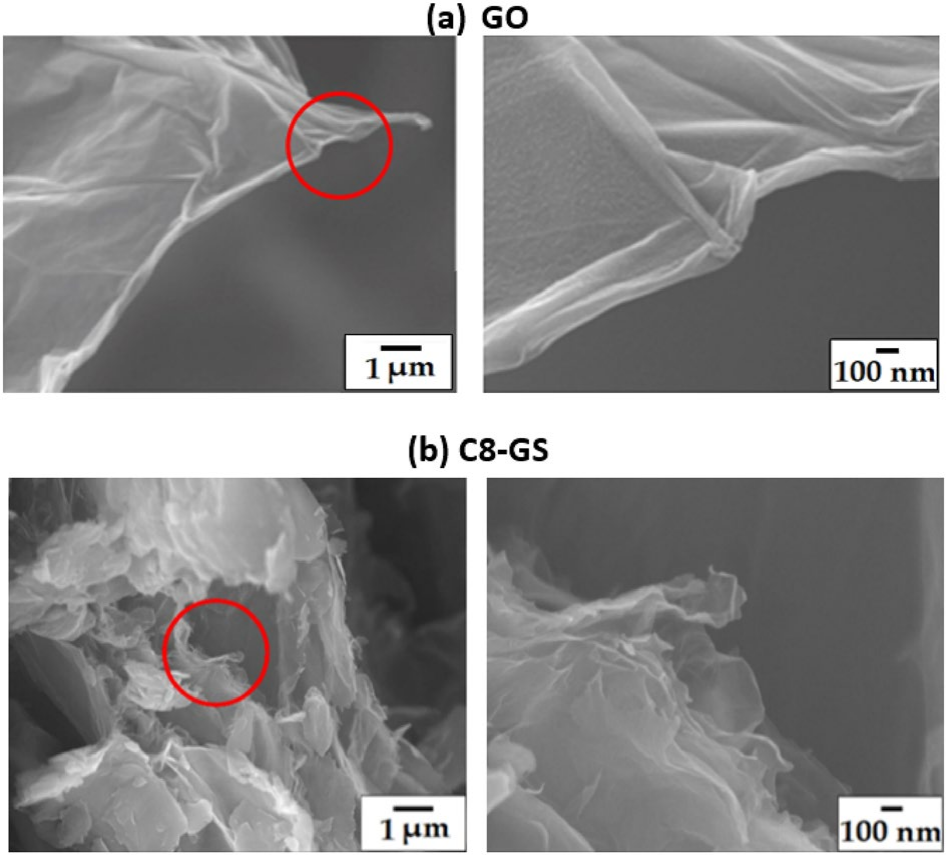
FE-SEM images of (**a**) GO and (**b**) C8-GS at magnifications of ×10,000 (left) and ×50,000 (right).

The FE-SEM images of GO and C8-GS are shown in Fig. 2. Although GO is partially folded and bent, it maintains the translucent plate shape and exhibits an intact layered structure (Fig. 2a). In the case of C8-GS, the clean form of GO is absent because the process of chemical modification of graphene with C8 severely damages the GS surface. A broken and wrinkled form is observed instead (Fig. 2b). F-GS substituted with a long alkyl group exhibits higher porosity, larger volume expansion, and lower density than pure GO (Fig. 2). To address this issue, a master batch is required when using the melt synthesis method^32^. However, these manufacturing disadvantages can be overcome if the polymer and F-GS are reacted together in solution.

Graphene is a planar carbon allotrope with a thickness of ~ 1 nm. However, it often agglomerates to form a multi-layered mass. Therefore, it is important to measure the actual thickness of graphene owing to the considerable difference between theoretical and actual values. AFM can be used to provide information about the properties of GS, such as defects, thickness, bending, and surface morphology.

**Figure 3:**
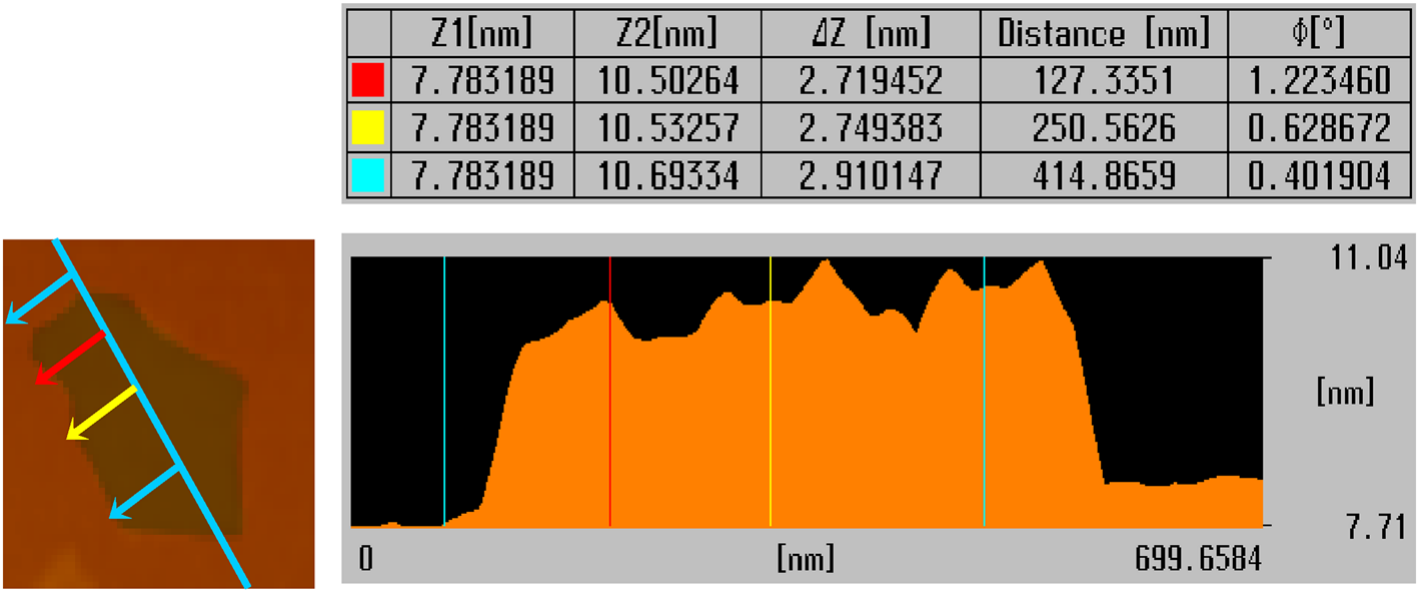
Non-contact-mode AFM images of C8-GS and line scan height profile along path indicated in images.

Stepped height scans allow the measurement of lateral size and thickness of monolayer graphene and F-GS. Figure 3 shows the AFM image of C8-GS obtained by depositing the dispersion on a mica substrate. The average thickness of the graphene layer in C8-GS treated with C8 was 2.79 nm. While the thickness of graphene is 1 nm, this discrepancy may arise from the chemical modification of C8 for pure GS.

### FT-IR and NMR spectra of PI

**Figure 4:**
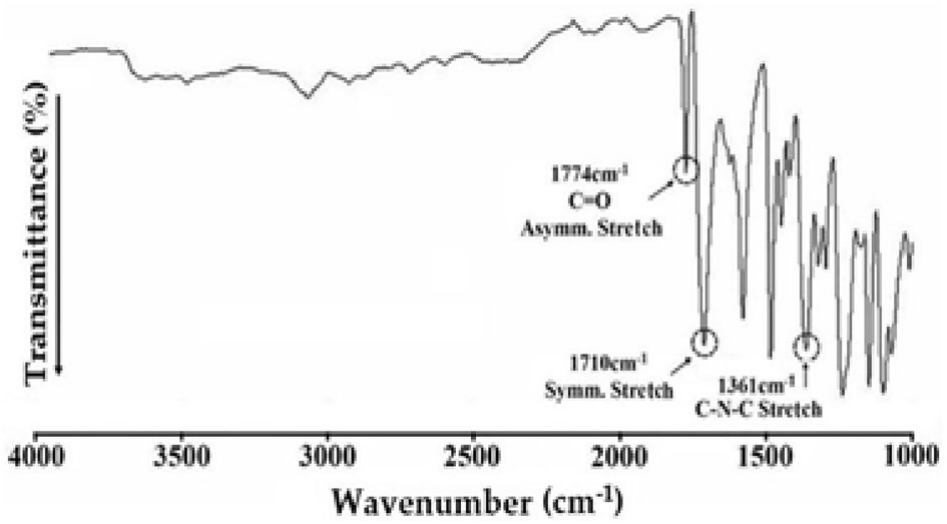
FT-IR spectrum of PI.

Figure 4 shows the FT-IR spectrum of the PI film synthesized using BPA and *m*-BAPS monomers. The C = O asymmetric and symmetric stretching peaks at 1774 cm^−1^ and 1710 cm^−1^, respectively, are noted. The characteristic C–N–C peak of the imide bond at 1361 cm^−1^ indicates that PI has been successfully synthesized^31^.

**Figure 5:**
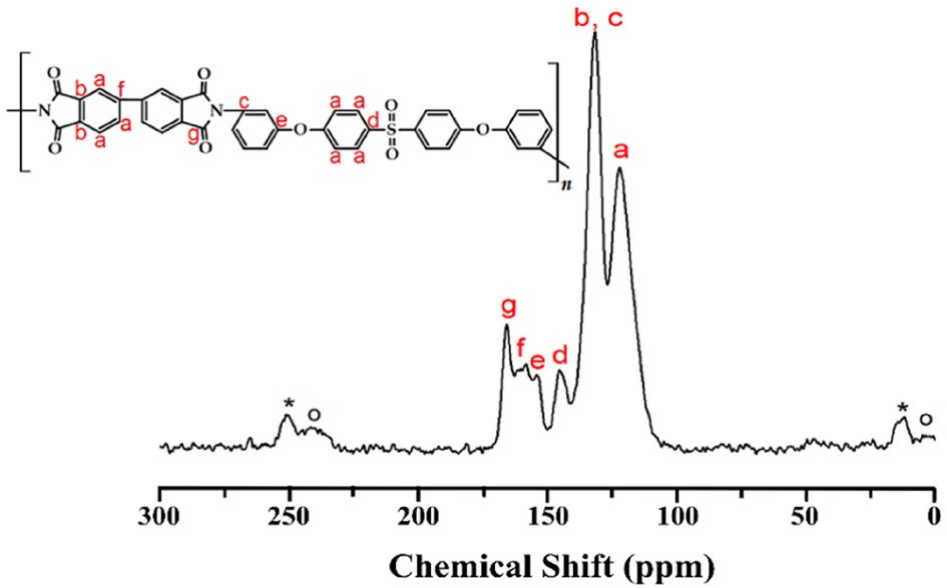
^13^C-NMR spectrum of PI.

In addition to FT-IR analysis, the structure of the as-synthesized PI was confirmed via solid-state ^13^C NMR spectroscopy (Fig. 5). The chemical shift values of ^13^C were calibrated to 0 ppm using the TMS standard peak at 38.3 ppm at 25 °C. The peaks corresponding to phenyl carbon (*a–f*) peaks are observed at 122.11, 131.78, 145.38, 154.13, and 158.49 ppm, respectively. The carbonyl (C=O, *g*) group of the imide group is observed at 165.92 ppm. The spinning sideband marked with an asterisk (*) is the sideband of peaks *b* and *c*, and the spinning sideband marked with an O is the sideband of peak *a*^33^.

### XRD patterns of the PI hybrid films

**Figure 6:**
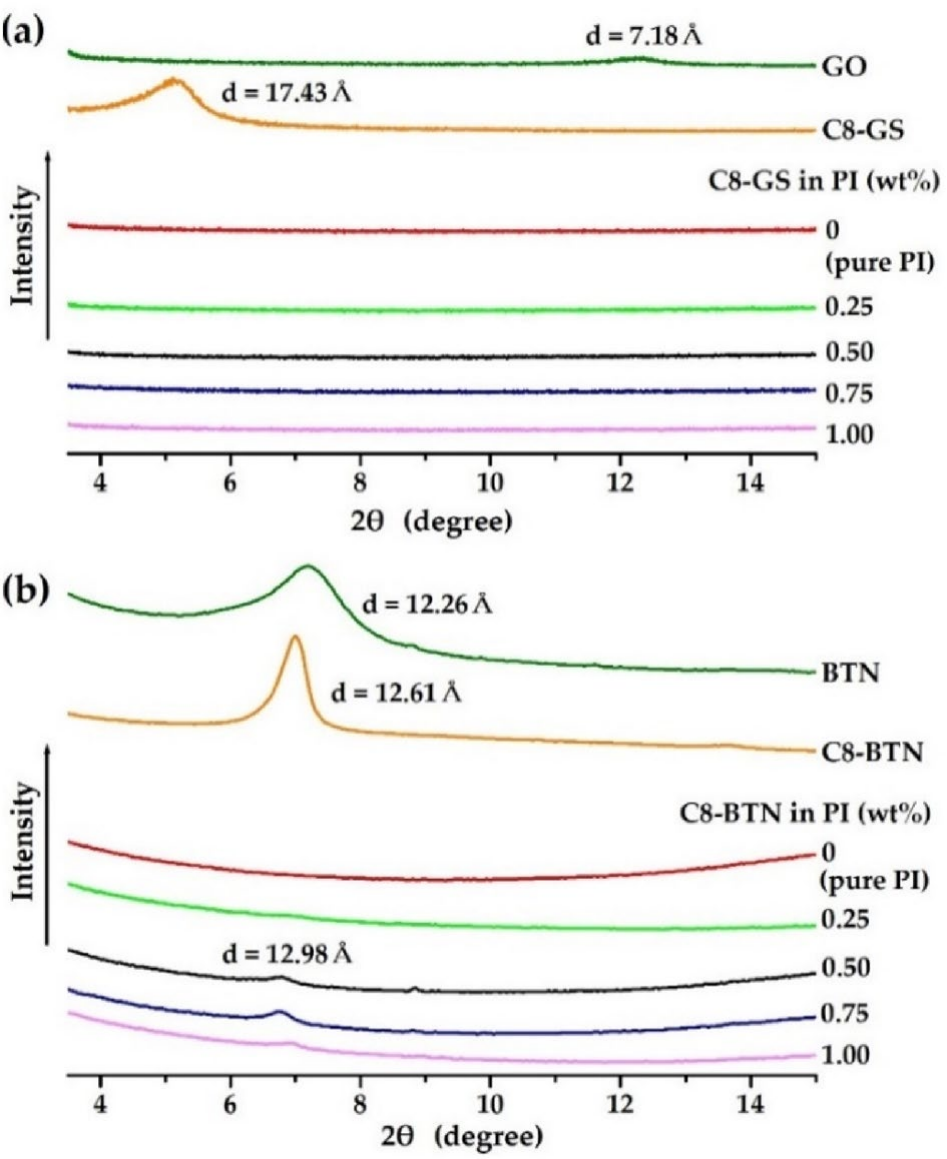
XRD patterns of the PI and PI hybrid films with different fillers. (**a**) C8-GS and (**b**) C8-BTN.

Figure 6 shows the XRD intensities against their respective *2θ* values in the range 3°–15° for GO, BTN, organically modified nanofillers, and CPI hybrid films containing various filler contents. While GO shows a weak diffraction peak at 12.30° (*d* = 7.18 Å) (Fig. 6a), a strong peak is observed at 5.07° (*d* = 17.43 Å) in C8-GS. The interlayer spacing obtained by replacing GO with C8 increased from 7.18 to 17.43 (*d* value); this can facilitate the insertion of polymer chains into the clay layer, thereby enhancing the dispersibility of the clay in the hybrid^22,34^. Even when C8-GS increases from 0 to 1.00 wt%, the peak of C8-GS is absent in the PI hybrid film (Fig. 6a). Thus, the C8-GS may be perfectly dispersed in the PI matrix, in the nanoscale without agglomeration.

The results of BTN, however, differ from those of GS. The diffracted characteristic peak of pure BTN appears at 7.46° (*d* = 12.26 Å), and a strong peak is observed at 7.00° (*d* = 12.61 Å) for the organoclay C8-BTN (Fig. 6b). This peak is present at nearly the same position (2*θ* = 6.80°, *d* = 12.98 Å) in all the hybrid films containing ≥ 0.50 wt% of C8-BTN, and its intensity gradually increases as the clay content increases to 1.00 wt%. Therefore, when a certain critical filler content is exceeded, the peak intensity and agglomeration of the clay also gradually increases^35,36^. However, the XRD patterns serve as only primary data to assess the degree of dispersion of the filler^37,38^. These results must be substantiated via electron microscopy, so as to observe the intercalation or exfoliation of the filler at the nanoscale.

### Morphologies of the PI hybrid films

**Figure 7:**
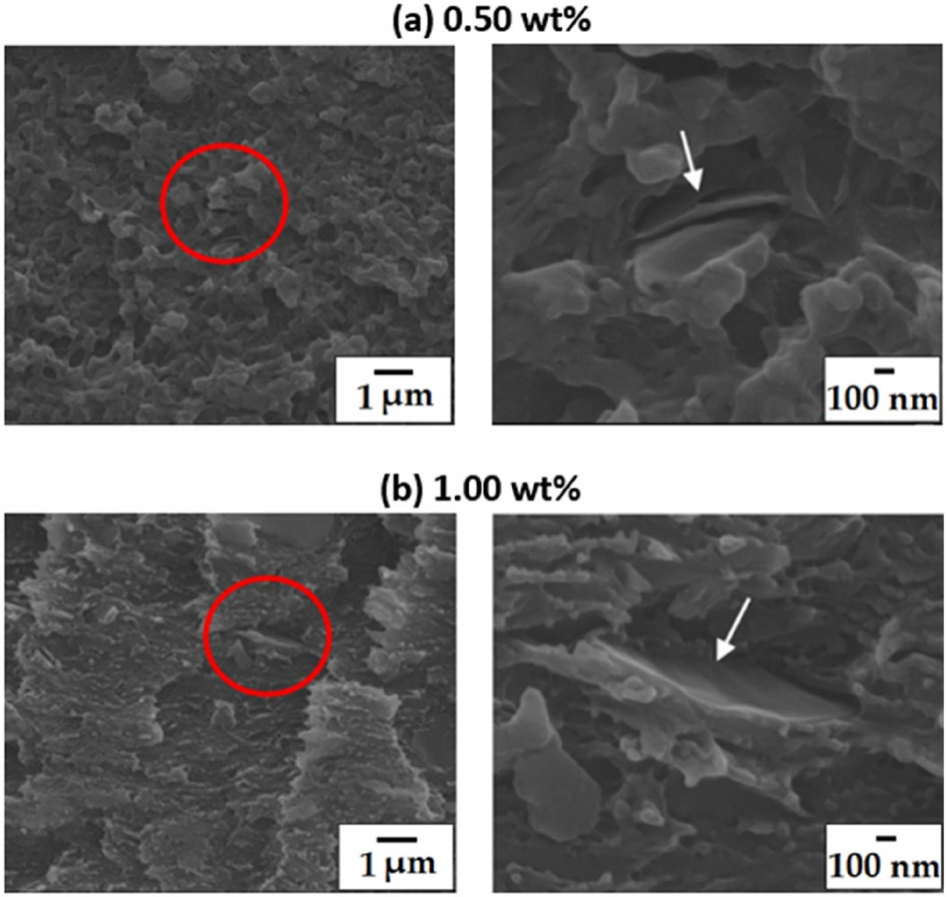
FE-SEM images of PI hybrid films with different C8-GS contents at magnifications of ×10,000 (left) and ×50,000 (right). (**a**) 0.50 wt% and (**b**) 1.00 wt%.

FE-SEM was used to observe the fracture shape of the PI hybrid films according to the C8-GS content (Fig. 7). For 0.50 wt% of C8-GS, the GS showed a straight plate shape in a certain direction (Fig. 7a). However, at 1.00 wt%, the GS is not dispersed, and instead aggregates (Fig. 7b). Thus, as the C8-GS content increases to 1.00 wt%, the degree of dispersion decreases, confirming that the graphene-films agglomerate better. In addition, the aggregation occurs readily beyond a critical C8-GS content.

**Figure 8:**
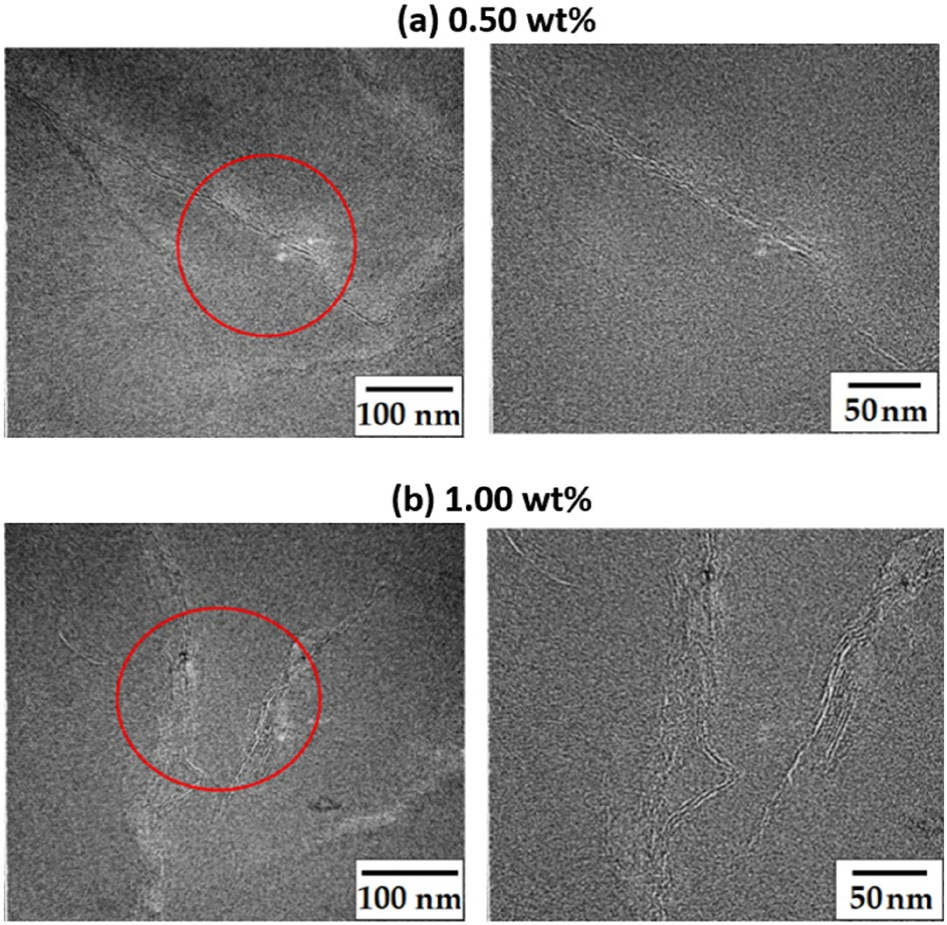
TEM images of the PI hybrid films with different C8-GS contents at magnifications of ×10,000 (left) and ×50,000 (right). (**a**) 0.50 wt% and (**b**) 1.00 wt%.

The TEM images show the exact dispersion of GS in the PI matrix. Figure 8 shows the TEM images of the hybrid films containing 0.50 and 1.00 wt% of C8-GS. The dark black line in the photo is the graphene layer, and the space between the dark lines is where PI exists. For 0.50 wt% C8-GS (Fig. 8a), the GSs are exfoliated in many areas in the polymer matrix. Although some agglomerate, they are usually dispersed with a size of < 30 nm. Even for 1.00 wt% C8-GS (Fig. 8b), the GSs are partially exfoliated from a large area of the polymer matrix, and a part is agglomerated, but micrometer-sized particles are absent. On an average, the aggregated GS particle size is less than ~ 50 nm, i.e., it is within the scale of a nanocomposite.

**Figure 9:**
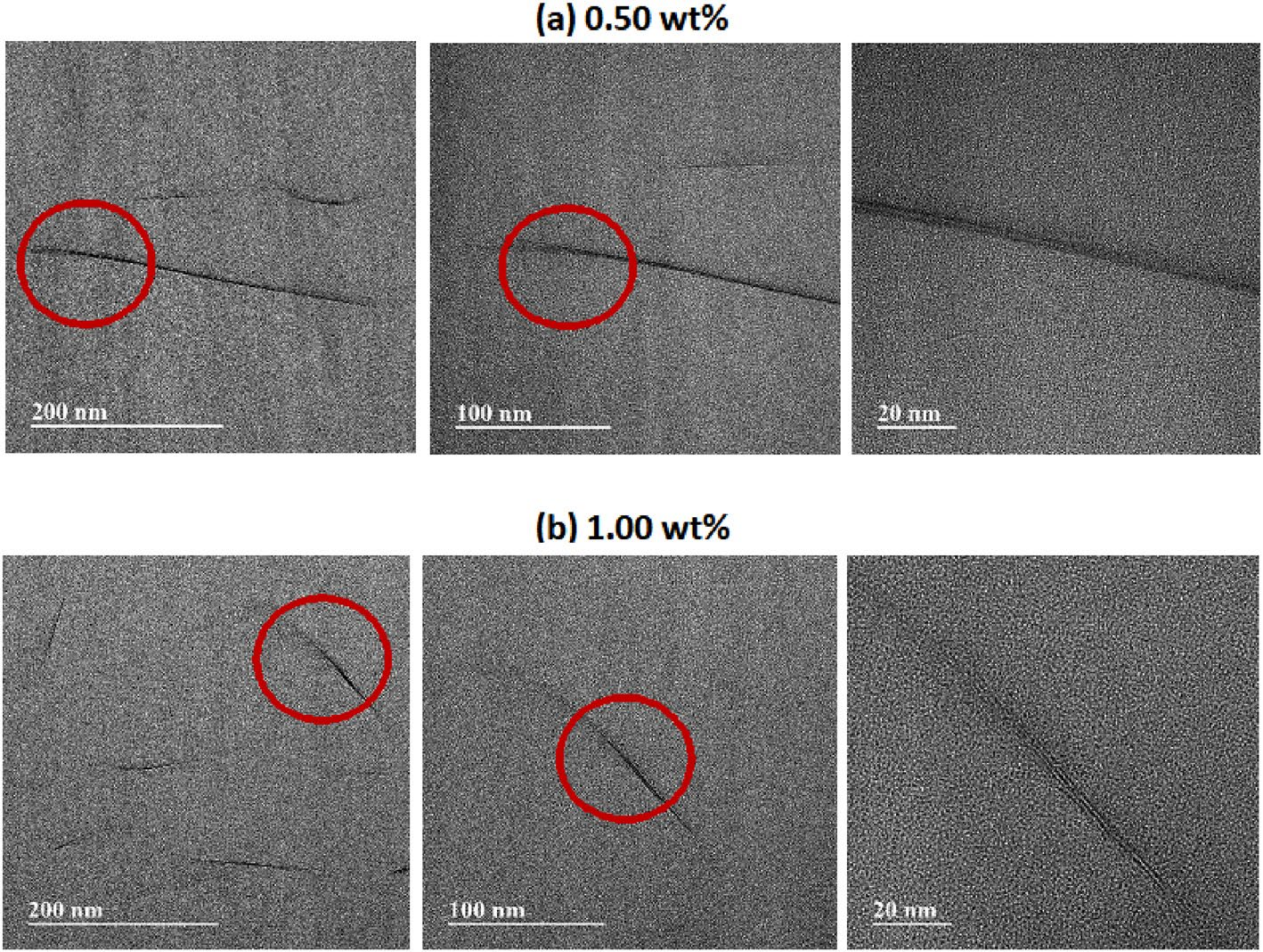
TEM images of the PI hybrid films with different C8-BTN contents of (**a**) 0.50 wt% and (**b**) 1.00 wt% at various magnifications.

C8-BTN (0.50 wt% BTN) forms a highly uniform dispersion (Fig. 9a). Most of the clays on the PI matrix show an exfoliated state and some are agglomerated, but the thickness is < 5 nm. Even for 1.00 wt% of C8-BTN, the structure is almost similar to that for 0.50 wt% C8-BTN. Thus, most of the clay is exfoliated, but agglomeration of ~ 10 nm is also observed in some parts (Fig. 9b).

Despite no considerable difference in the dispersion state of GS and clay for PI noted from the TEM images, clay disperses better than GS. This can influence the thermal properties and optical transparency of the resulting hybrid, which is discussed later.

### Thermal properties of PI hybrid films

**Figure10:**
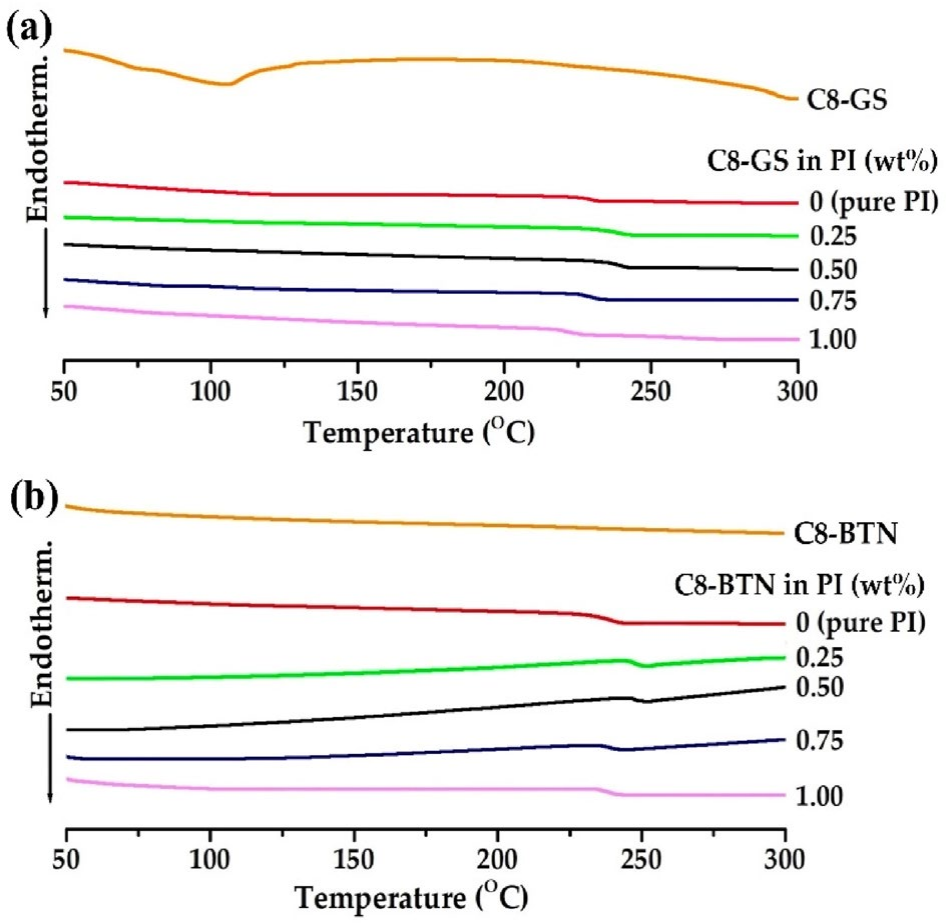
DSC thermograms of the pure PI and PI hybrid films with different fillers. (**a**) C8-GS and (**b**) C8-BTN.

Since PIs are mostly amorphous polymers, the melting transition temperature (*T*_*m*_) is not observed via DSC, and the material characteristics are mainly explained by the glass transition temperature (*T*_*g*_). The *T*_*g*_ of a polymer varies depending on structural differences, chemical bonds such as hydrogen bonding and those formed during curing reactions, chain fluidity according to free volume, and presence of additives^39^. Table 2 shows enlists the thermal properties of the PI hybrid with varying nanofiller content. In the case of aromatic PI, the fluidity of the polymer chain is poor and generally has a high *T*_*g*_. The *T*_*g*_ of pure PI is 225 °C, but the *T*_*g*_ of C8-GS (up to 0.50 wt%) is increased by 8 °C (to 233 °C) compared to pure PI. This can be attributed to the GS being dispersed in the matrix polymer and disrupting the segmental movement of the polymer chain by changing the free volume. The GS nanofiller layers may form rigid plates, disturbing the movement of the polymer chains between them; the segmentation motion of the polymer chains becomes difficult and the *T*_*g*_ increases^40,41^. However, when the concentration of C8-GS is increased to 1.00 wt%, the *T*_*g*_ of the hybrid film decreases to 218 °C. Similar results were obtained when BTN was used as a filler. The PI hybrid, C8-BTN (0.50 wt%) shows the highest *T*_*g*_ of 246 °C, which decreases to 234 °C when 1.00 wt% of the organoclay is used. When the fillers added to the hybrid are above a certain critical concentration, they are not dispersed, but rather aggregated with each other, which reduces the *T*_*g*_. The aggregation of the filler above a certain critical concentration is substantiated by electron microscopy results described previously (Figs. 7, 8, 9), and corroborate with the results of previous studies^42,43^. Figure 10 shows the DSC thermograms of the PI hybrids according to the various contents of the two types of nanofillers.

**Table 2:** Thermal properties of the PI hybrid films with different fillers.

Filler in PI (wt%)	C8-GS				C8-BTN			
	*T*_*g*_ (°C)	*T*_*D*_^*i*a^ (°C)	*wt*^*600*b^ (%)	CTE^c^ (ppm/°C)	*T*_*g*_ (°C)	*T*_*D*_^*i*a^ (°C)	*wt*^*600*b^ (%)	CTE^c^ (ppm/°C)
0 (pure PI)	225	278	73	48.9	225	278	73	48.9
0.25	229	407	70	47.0	244	456	69	42.3
0.50	233	452	70	46.9	246	504	71	41.7
0.75	224	446	71	47.6	235	478	72	39.7
1.00	218	299	71	47.8	234	476	65	39.3

^a^Initial decomposition temperature at 2% weight loss. ^b^Weight residue at 600 °C.^c^Coefficient of thermal expansion for 2nd heating is 50 ~ 200 °C.

**Figure 11:**
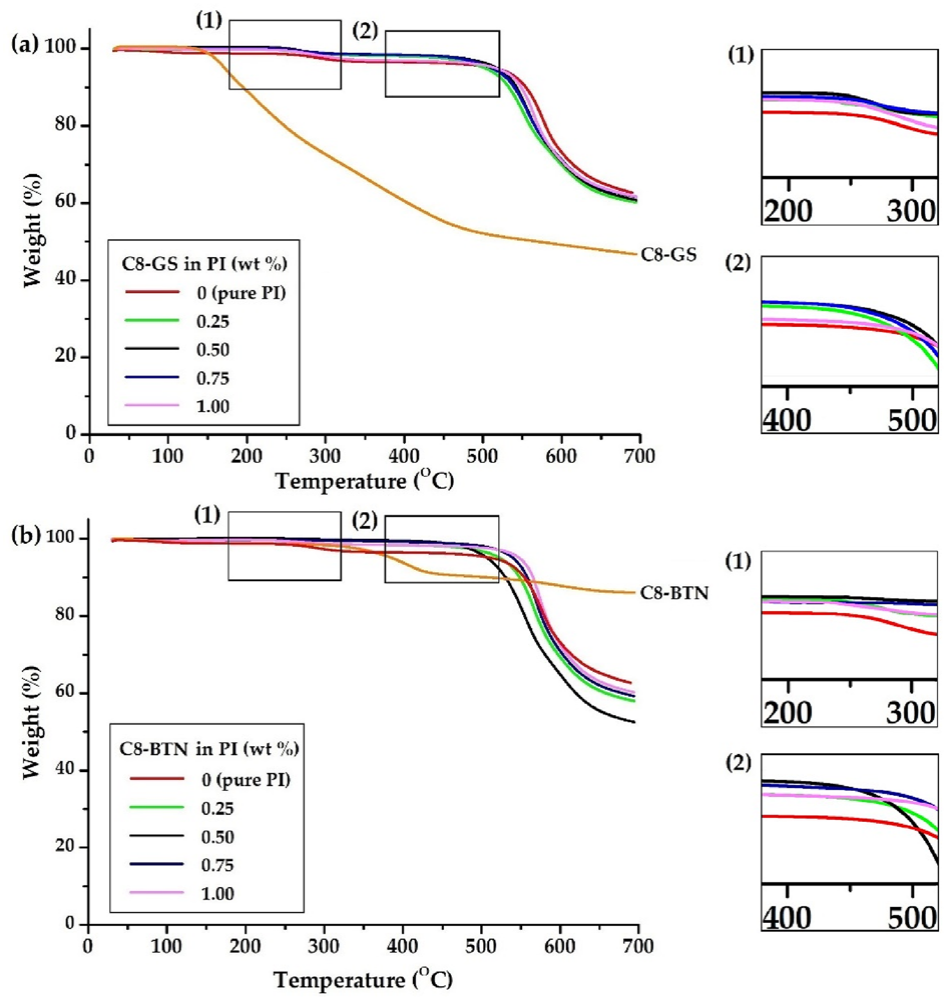
TGA thermograms of the pure PI and PI hybrid films with different fillers. (**a**) C8-GS and (**b**) C8-BTN.

Figure 11 shows the thermograms of the two types of nanofillers. The initial decomposition of C8-GS and C8-BTN already began to appear around 150 °C due to octylamine, which has very low thermal stability. In particular, for C8-GS synthesized using GO, a significant amount of decomposition occurred owing to the functional groups contained in GO (see Supplementary Information S1).

Table 2 shows the thermal stability of the PI hybrid film using various amounts of nanofillers. As noted in the *T*_*g*_ results, the initial decomposition temperature (*T*_*D*_^*i*^) increases with increasing filler content in the hybrid until a certain critical filler concentration is reached, beyond which *T*_*D*_^*i*^ tends to decrease. For example, the *T*_*D*_^*i*^ of pure PI is 278 °C; for 0.50 wt% of fillers in C8-GS and C8-BTN, the *T*_*D*_^*i*^ of the hybrids increased rapidly to 452 °C and 504 °C, respectively. This can be attributed to the excellent thermal stability and dispersibility of the fillers in the polymer matrix at concentrations of up to 0.50 wt%, which suppresses the thermal decomposition of the polymer, and simultaneously prevents the outflow of volatilized gaseous components^44,45^. However, when the content of the two fillers is increased to 1.00 wt%, the thermal decomposition inhibitory effect does not appear due to the aggregation of the fillers, and the *T*_*D*_^*i*^ decreases. In the case of C8-GS and C8-BTN, it decreases to 299 °C and 476 °C, respectively. Thus, the aggregation of fillers reduces the thermal stability above a certain critical concentration, as confirmed by the *T*_*g*_ values. On heating to 600 °C, the residue weight value (*wt*_*R*_^*600*^) is 73% for pure PI, but shows an almost constant value of 70% when the filler content is 0.25–1.00 wt%. The reason for the consistently high *wt*_*R*_^*600*^ values is the excellent heat resistance of the plate layers of GS and BTN, which are well dispersed even after the substituted alkyl group is decomposed at a high temperature. Figure 11 shows the TGA thermograms of the hybrid films according to various filler contents. In all the TGA thermograms of, a small weight loss is observed at ~ 200 °C due to the decomposition of C8 (see Supplementary Information S1), and the full-scale thermal decomposition of PI starts at ~ 450 °C.

Overall, the C8-BTN hybrid shows superior thermal properties than C8-GS. Because the aspect ratio of BTN (~ 218) is smaller than that of GS (> 250), it is effectively dispersed in the polymer matrix and enhances its various thermal properties. Further, the *T*_*D*_^*i*^ of C8-GS and C8-BTN is 157 °C and 324 °C, respectively; thus, the thermal stability of organoclay is higher than that of F-GS (Fig. 11; Supplementary Information S1).

**Figure 12:**
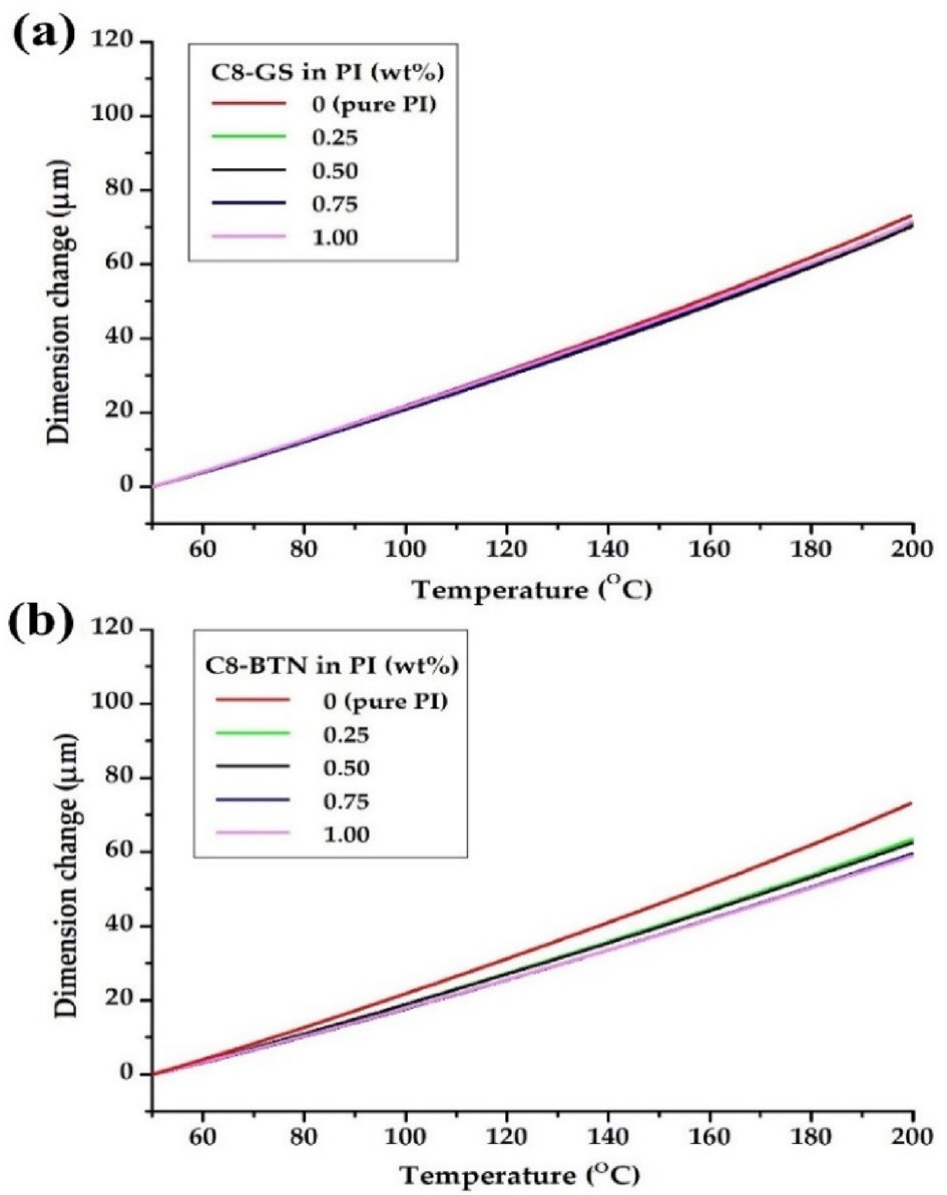
TMA thermograms of the pure PI and PI hybrid films with different fillers. (**a**) C8-GS and (**b**) C8-BTN.

When the pure polymer is heated, it relaxes in a perpendicular direction to the PI main chain. However, for hybrids with layers of rigid and strong, plate-like GS or BTN, heat-induced deformation is difficult. Therefore, graphene or clay, which have high thermal stabilities and can efficiently block heat transfer, can suppress the heat-induced lateral thermal expansion of polymers^46–48^. In conclusion, in order for the hybrid to have low thermal expansion characteristics, the polymer matrix and filler must be thermally stable. Table 2 lists the CTE values of the PI hybrid films with various nanofiller loadings obtained after a secondary heating in the temperature range 50–200 °C, and their TMA thermograms are shown in Fig. 12. In the C8-GS hybrid, the CTE values are nearly constant between 46.9 and 47.8 ppm/°C, regardless of the filler content. However, in C8-BTN, when the filler content is 0.25%, the CTE decreases from 48.9 to 42.3 ppm/°C; as the filler content increases to 1.00 wt%, the CTE value shows an almost constant value between 39.3 and 41.7 ppm/°C.

### Mechanical properties of PI hybrid films

Similar to the critical concentration of fillers observed for thermal properties, nanofillers had a significant effect on mechanical properties. Table 3 summarizes the mechanical tensile properties of the PI hybrid films containing various nanofiller contents. For example, in the case of the C8-GS hybrid, when the C8-GS content increased from 0 to 0.5 wt%, the tensile strength increased from 54 to 81 MPa. However, when the C8-GS content increased to 1.00 wt%, the ultimate tensile strength decreased to 66 MPa. This result can also be explained by the agglomeration of GS at critical concentration. The C8-BTN hybrid showed a similar trend to that of C8-GS. The final tensile strength of pure CPI was 54 MPa, but when the concentration of C8-BTN reached 0.50 wt%, the value increased by 174% (95 MPa). However, the tensile strength decreased to 85 MPa when the clay content increased to 1.00 wt%. Similar reports have been published for polymer hybrid systems from many other groups, including our research group. We have reported similar results in PI hybrids containing functionalized graphene and organoclays^28^. Yano et al.^49^ also reported that the mechanical properties of nanocomposites decreased above a critical concentration of silica, explaining that the filler particles did not evenly disperse and agglomerate with one another above the critical concentration. In this study, we confirmed this phenomenon using TEM images, as shown in Figs. 8 and 9.

**Table 3:** Mechanical properties of the PI hybrid films with different fillers.

Filler in PI (wt%)	C8-GS			C8-BTN		
	Ult. Str. (MPa)	Ini. Mod. (GPa)	EB (%)	Ult.Str. (MPa)	Ini. Mod. (GPa)	EB (%)
0 (pure PI)	54	3.52	7	54	3.52	8
0.25	62	4.89	7	73	5.09	8
0.50	81	5.24	6	94	5.81	7
0.75	69	6.09	5	90	7.05	9
1.00	66	7.12	5	85	7.87	8

^a^Ultimate strength, ^b^Initial modulus, ^c^Elongation at break.

However, unlike the ultimate tensile strength, the initial tensile modulus increased steadily in proportion to the amount of graphene and clay (Table 3). In the two series of C8-GS and C8-BTN, the initial modulus gradually increased from 3.52 GPa to 7.12 and 8.08 GPa, respectively, when the content of nanofillers increased from 0 to 1.00 wt%. The increase in the initial tensile modulus of the two hybrid series can be explained by the type of filler (rigid rod), the high aspect ratio and the directionality of the clay and graphene layers, and the resistance of the filler itself to external forces^50,51^.

Over the range of filler contents, the elongation at break (EB) of the C8-GS PI and C8-BTN PI hybrids was 5–7% and 7–9%, respectively; thus, there was no significant difference between the two series (see Table 3). The EB value of our study was lower than that of other polymer hybrids.These results are typical of hybrid materials reinforced with hard but brittle inorganic materials ^51^.

When the thermo-mechanical properties of the PI hybrid containing C8-GS and C8-BTN as fillers were compared, although the interlayer distance of the clay layer of C8-BTN synthesized using octylamine did not change significantly compared with that of pure BTN (Fig. 6b), the overall thermo-mechanical properties were better than that of pure PI and C8-GS hybrids. These results are presumed to be excellent dispersibility and compatibility of C8-BTN and PI matrix. In addition, as described above, the dispersion of clay with a smaller aspect ratio than graphene also played a major role in increasing the physical properties.

### Optical transparencies of the PI hybrid films

**Figure 13:**
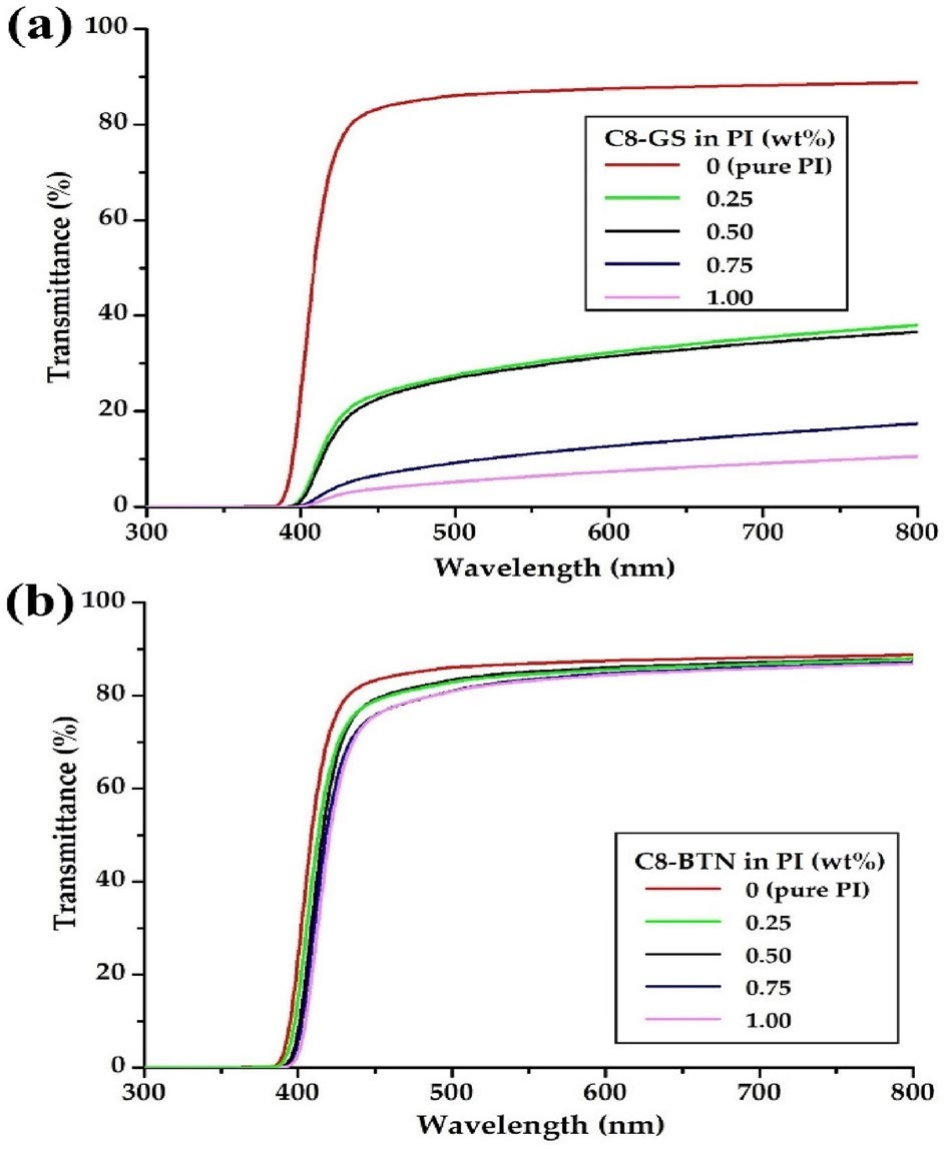
UV–Vis transmittances of the pure PI and PI hybrid films with different fillers. (**a**) C8-GS and (**b**) C8-BTN.

To reduce the error in measuring the optical properties of the hybrid films, films of thickness 44–50 μm were prepared. Table 4 summarizes the optical transparency results of the PI hybrid films according to the two types of fillers of varying contents. The optical properties of CPI films can be assessed in terms of their λ_o_, transmittance at 500 nm (500_nm_^trans^), and YI. Pure PI films are colorless and transparent because molecular interactions between the bent polymer chains is unlikely; thus, CTCs are not formed. An increase in the filler content is accompanied by an increase in the λ_o_ value of the PI hybrid film, and a decrease in the 500_nm_^trans^ because the filler particles aggregate with each other. For example, in the C8-GS hybrid, λ_o_ steadily increases from 384 to 398 nm with increasing filler content from 0 to 1.00 wt%, but the value of 500_nm_^trans^ decreases sharply from 86 to 5%. Similarly, in the C8-BTN hybrid, λ_o_ increases from 384 to 394 nm with increasing filler content from 0 to 1.00 wt%, and the 500_nm_^trans^ slightly decreases from 86 to 81%. Figure 13 shows the UV–Vis. transmittances according to the various contents of the two types of fillers. The YI value of the hybrid film increases from 4 to 37 when the C8-GS content increases to 1.00 wt%, but maintains a constant value of YI = 5 regardless of the filler content in the C8-BTN hybrid.

**Table 4:** Optical properties of the PI hybrid films with different fillers.

Filler in PI (wt%)	C8-GS				C8-BTN			
	Thickness (μm)	λ_0_^a^ (nm)	500nm^trans^ (%)	YI^b^	Thickness (μm)	λ_0_ (nm)	500nm^trans^ (%)	YI
0 (pure PI)	50	384	86	4	40	384	86	4
0.25	48	392	27	23	40	386	83	5
0.50	44	393	26	23	44	390	83	5
0.75	50	394	9	25	44	392	81	5
1.00	49	398	5	37	45	394	81	5

^a^Cut-off wavelength. ^b^Yellow index.

**Figure 14:**
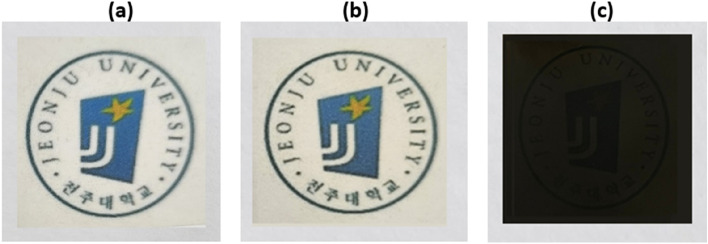
Photographs of the PI hybrid films with different fillers. (**a**) 0 wt% (pure PI), (**b**) 0.50 wt% C8-BTN, and (**c**) 0.50 wt% C8-GS.

Photographs of the films placed on the Jeonju University logo are presented in Fig. 14 to visually check the transparency of the PI hybrid film synthesized in our laboratory. The pure PI film itself is not completely colorless and transparent and has a slight yellowish color (Fig. 14a); thus, the difference in yellowness between the C8-BTN hybrid and pure PI cannot not be distinguished even when the filler content is increased by 0.50 wt% (Fig. 14b). The letters of the logo are clearly visible, and the film does not become cloudy, or retains transparency even when the organoclay content is increased to 1.00 wt%. Contrarily, 0.50 wt% of C8-GS dispersed in the PI film appears completely black and the underlying logo cannot be seen (Fig. 14c). These results do not change until the filler content is raised to 1.00 wt%.

When using the same amount of filler, the PI hybrid with organoclay has a better optical transparency than that using functionalized-graphene. This is because graphene not only deteriorates the optical properties of the film due to its inherently black color, but also causes the particles to agglomerate with each other when excessively used in hybrids.

## Conclusion

We observed the properties of the hybrid films upon varying the types and contents of different nanofillers for the PI matrix polymer. The thermal, morphological, and optical properties of the hybrid films obtained by using fillers of functionalized graphene (C8-GS) and organoclay (C8-BTN), at contents of 0.25–1.00 wt% in the PI matrix, were compared. *T*_*g*_ and *T*_*D*_^*i*^ have the highest values when the filler content is 0.50 wt% in both the hybrids, but decrease above the critical concentration. Overall, the thermal and optical properties of the C8-BTN hybrid are superior to those of the C8-GS hybrid; this is closely related to the morphology of the fillers dispersed in the matrix.

As a type of super-engineering plastics, PIs serve as a high-performance material that can be used under harsh conditions owing to its excellent thermomechanical properties, chemical resistance, and optical transparency; PIs with excellent physicochemical properties can be synthesized by accurately designing the structure of the monomer and controlling the reaction conditions. The uniform, nanoscale dispersion of fillers in the PI matrix using a propellant designed to have excellent dispersibility and high interfacial adhesion, produces nanocomposites with excellent physical properties that cannot be obtained via conventional manufacturing processes. These improved nanocomposites can be applied as film materials in the electrical, electronic, and optical fields.

## Supplementary Information

Supplementary Figure 1.

## Data Availability

The datasets used and/or analysed during the current study available from the corresponding author on reasonable request.
