# Supplementary figures and images for: Comparison of the properties of polyimide nanocomposite films containing functionalized-graphene and organoclay as nanofillers
Source: Sci Rep. 2022 Dec 3;12:20892. doi: 10.1038/s41598-022-25178-2 (PMC9719546; doi:10.1038/s41598-022-25178-2)

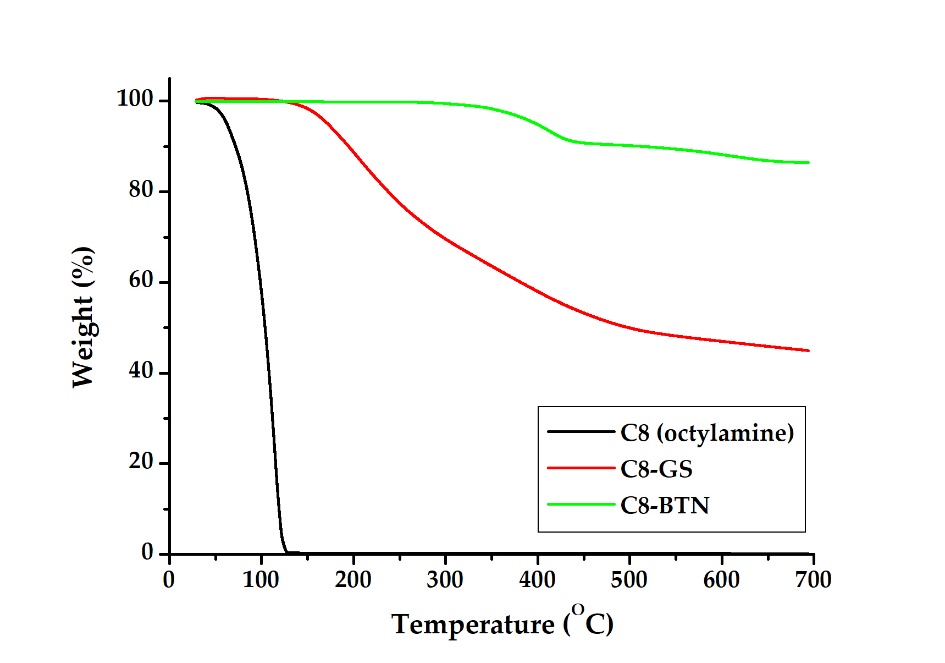


**Supplement-Figure S1.** TGA thermograms of C8, C8-GS, and C8-BTN.

Supplement: Supplementary file 1 — Supplementary Figure 1. [file 41598_2022_25178_MOESM1_ESM.docx]
